# A New Laboratory Tool for COVID-19 Severity Prediction, CENIL Score

**DOI:** 10.3390/diagnostics14222557

**Published:** 2024-11-14

**Authors:** Elif Mukime Saricaoglu, Belgin Coskun, Muge Ayhan, Esragul Akinci, Bircan Kayaaslan, Adalet Aypak, Ayse Yasemin Tezer Tekce, Imran Hasanoglu, Ayse Kaya, Fatma Eser, Yesim Aybar Bilir, Burcu Ozdemir, Turan Buzgan, Rahmet Guner

**Affiliations:** 1 Department of Infectious Diseases and Clinical Microbiology, Faculty of Medicine, Ankara University, Ankara 06100, Turkey; 2 Department of Infectious Diseases and Clinical Microbiology, Ankara City Hospital, Ankara 06800, Turkey; belgintekin@yahoo.com (B.C.); dr.mugeayhan@hotmail.com (M.A.); yesimaybar@gmail.com (Y.A.B.); burcubagci17@hotmail.com (B.O.); 3 Department of Infectious Diseases and Clinical Microbiology, Ankara City Hospital, University of Health Sciences, Ankara 06290, Turkey; esragulakinci@gmail.com (E.A.); aadalet@gmail.com (A.A.); ayasmintezer@gmail.com (A.Y.T.T.); 4 Department of Infectious Diseases and Clinical Microbiology, Ankara City Hospital, Ankara Yıldırım Beyazıt University, Ankara 06690, Turkey; drbican@gmail.com (B.K.); imransolak@gmail.com (I.H.); dr.aysekaya09@hotmail.com (A.K.); fatmacivelekeser@hotmail.com (F.E.); turanbuzgan@aybu.edu.tr (T.B.); hrguner@aybu.edu.tr (R.G.)

**Keywords:** COVID-19, disease severity, risk score, laboratory parameter

## Abstract

Background/Objectives: Several studies investigated the risk factors for severe COVID-19-related outcomes. Early identification and proper treatment of COVID-19 patients who may develop severe pneumonia are crucial. The aim of this study was to detect the importance of the laboratory parameters for risk prediction of severe pneumonia in COVID-19 patients. Methods: This retrospective cohort study included COVID-19 patients’ laboratory parameters at admission. Biochemical, hematological, coagulation, and inflammatory parameters values were compared between the non-severe and severe groups. Results: A total of 534 COVID-19 patients were screened, and 472 of them were included in this study. The mean age of patients was 64 (±3.1) years; 242 (51.3%) were men. A total of 204 (43.2%) patients were diagnosed as severe cases. The independent predictors of severe illness were C-reactive peptide, Eosinophil, neutrophil–lymphocyte ratio, interleukin-6, and lactate dehydrogenase. These parameters were named as CENIL scores from 0 to 5 points. The findings of this study indicate that these biomarkers identified tend to increase progressively with disease severity in severe COVID-19 patients. Additionally, the CENIL risk score identified a specific cut-off value of 3, highlighting it as a critical threshold for identifying patients at high risk of severe COVID-19 progression. Conclusions: In this study, we identified biomarkers—including CRP, eosinophil count, NLR, IL-6, and LDH—named as CENIL risk score that can help predict the likelihood of severe disease at diagnosis. Clinicians may be more vigilant regarding the development of severe disease in patients with high CENIL risk scores, guided by clinical and radiological findings.

## 1. Introduction

The COVID-19 pandemic become a global threat to human health [1]. Despite the most common symptoms being cough, fatigue, fever, and myalgia, the clinical spectrum of COVID-19 is quite wide, from asymptomatic/mild infection to severe pneumonia and death [2,3]. For the latter phase of the disease, COVID-19 patients can be presented with hyperinflammatory syndrome or cytokine storm characterized by dysregulated and systemic immune overactivation. These patients quickly progress into respiratory failure or multiple organ dysfunction syndrome. For this reason, early identification of these critical patients is very important [4].

There are several studies investigating the risk factors for severe COVID-19-associated outcomes [1]. Some of these studies on COVID-19 have created several types of prediction models using laboratory parameters as the inputs [5,6,7]. The SARS-CoV-2 has been shown in multiple tissues, including lung, endothelial, renal, and neuronal cells. The invasion of different tissues and organs may help explain the mechanism that causes the systemic effects of the virus. Therefore, laboratory biomarkers related to organ damage can be used for the detection of patients at high risk for severe disease [8]. Several biomarker scores are feasible in COVID-19. Some of these can predict disease prognosis and outcomes [9]. Higher levels of IL-6, ferritin, D-dimer, C-reactive peptide (CRP), and liver enzymes are associated with critical illness [5,7,10,11]. Birlutiu et al. showed that various biomarkers, notably CRP, LDH, D-dimers, and ferritin, show a progressive rise with increasing disease severity and are notably elevated in those with poor outcomes. The differences observed between severity levels and outcome groups are statistically significant, highlighting the prognostic importance of these biomarkers in older adults [12]. In recent studies on COVID-19, various biomarkers have been identified as predictors of unfavorable patient outcomes, falling into several categories: hematological parameters, inflammatory markers, lipid and glucose profiles, iron metabolism markers, coagulation markers, and indicators of organ dysfunction. Each of these biomarkers highlights a specific aspect of COVID-19’s impact on the body, offering valuable insights into the disease’s pathology. By analyzing these laboratory parameters, clinicians can better tailor therapeutic strategies, aligning treatment methods with the patient’s unique disease profile [13]. Salton et al. found that high levels of cytokines between days 7 and 14 of hospitalization in COVID-19 patients with acute respiratory distress syndrome (ARDS) who were on glucocorticoid treatment and non-invasive ventilation (NIV) were linked to NIV failure and an increased need for invasive mechanical ventilation (IMV). The IMV group showed higher inflammation levels at intubation, which may indicate glucocorticoid resistance, so higher glucocorticoid doses could serve as a better agent for inflammation control and outcome in these patients. The cytokine patterns could be used as early prognostic markers to optimize the timing of IMV initiation [14].

Early identification and proper treatment of COVID-19 patients who may develop severe pneumonia is very crucial. In our study, characteristics of biochemical, hematological, coagulation, and inflammatory parameters of COVID-19 patients were investigated, which help to apply personalized management. The aim of this study is to develop a risk-scoring system based on laboratory parameters predictive of disease severity to estimate COVID-19 progression. This scoring system would integrate key biomarkers to offer a reliable assessment tool for predicting clinical outcomes and guiding treatment decisions.

## 2. Materials and Methods

This retrospective cohort study included patients followed at the main pandemic hospital in the capital city of Türkiye. A confirmed case is defined as a person with reverse transcriptase polymerase chain reaction (RT-PCR) test confirmation of COVID-19 infection with the clinical signs and symptoms. All COVID-19 patients diagnosed between 15 September–15 December 2020 were screened. All patients met the following inclusion criteria: 1. adult patients (≥18 years old), 2. routine laboratory testing (biochemical, hematological, coagulation, and inflammatory parameters) at admission, 3. RT-PCR test positive for SARS-CoV-2. Patients meeting the following criteria were excluded: (1) those still hospitalized before 15 December 2020; (2) leaving follow-up; (3) missing baseline laboratory data; (4) using corticosteroid due to primary diseases; (5) having splenectomy operation/functional asplenia; (6) hematological malignancy; (7) pregnancy.

Clinical syndromes associated with COVID-19 were classified as mild illness, pneumonia, severe pneumonia, acute respiratory distress syndrome, and sepsis/septic shock according to WHO case definitions [10]. Patients who had a fever or suspected respiratory infection, plus one of the following: respiratory rate over 30 breaths per minute, severe respiratory distress, or partial oxygen saturation ≤ 93% on room air, were described as having severe pneumonia. For comparisons, patients were categorized into two groups: non-severe group (asymptomatic/mild illness/pneumonia) and severe group (severe pneumonia/ARDS). Various parameters of peripheral blood routines of COVID-19 patients were recorded on the day of admission. Biochemical (glucose, creatinine, total protein, albümin, creatinine phosphokinase (CPK), aspartate transaminase (AST), alanine transaminase (ALT), total bilirubin, lactate dehydrogenase (LDH), troponin-I), hematological (white blood cell count, neutrophil count, lymphocyte count, eosinophil count, monocyte count, platelet count, hemoglobin, neutrophil/lymphocyte ratio (NLR)), coagulation (active partial thromboplastin time (aPTT), prothrombin time (PTT), INR, fibrinogen, D-dimer), and inflammatory parameters (C-reactive peptide (CRP), procalcitonin, ferritin, interleukin-6 (IL-6) values were compared between non-severe and severe groups.

This study was approved by the Ethics Commission of Ankara City Hospital (No: 1/1559/2021). Informed consent for our retrospective observational study was obtained from patients.

Statistical analysis was performed using SPSS software (Version 23, IBM SPSS., Armonk, NY, USA). The variables were investigated using visual (histograms, probability plots) and analytical methods (Kolmogorov–Smirnov/Shapiro–Wilk’s test) to determine whether or not they are normally distributed. Descriptive analysis was presented using means and standard deviations for normally distributed variables, median (min–max) for non-normally distributed variables, and frequencies for ordinal variables. The significance of the difference between the groups in terms of mean and median were investigated, respectively, with Student’s *t*-test and Mann–Whitney test. The Fisher’s exact test or Chi-square test, where appropriate, was used for comparing categorical variables in different groups. The parameters affecting the group variables were evaluated with univariate analysis, after which the parameters with *p* < 0.05 were included in the multivariate logistic regression analysis. Independent risk factors were determined. The capacity of laboratory parameters to predict severe disease in COVID-19 patients was analyzed using Receiver Operating Characteristics (ROC) curve analysis. Cut-off values for discriminative laboratory parameters were calculated according to Youden index. When a significant cut-off value was observed, the sensitivity, specificity, and positive and negative predictive values were assessed. For the multivariate analysis, the possible factors identified with univariate analyses were further entered into the logistic regression analysis to determine independent predictors of severe disease. The odds ratio and confidence interval of the statistically significant variables were presented. Severe disease risk score (CENIL) was constructed based on these variables from the logistic model. The accuracy of the CENIL risk score was assessed using the area under the receiver–operator characteristic curve (AUC). A *p*-value of less than 0.05 was considered to show a statistically significant result.

## 3. Results

A total of 534 patients diagnosed with COVID-19 between 15 September and 15 December 2020 were identified. Finally, a total of 472 eligible patients were included in this study. The mean age of patients in the cohort was 64 (±3.1) years; 242 patients (51.3%) were men, 334 (70.7%) had at least one coexisting condition, including 235 (49.8%) with hypertension, 165 (35%) with diabetes, and 100 (21.2%) with coronary artery disease as the top the comorbidities (Table 1). Of these patients with COVID-19, 204 (43.2%) were diagnosed as severe cases. The overall mortality was 5.5%.

A comparison of biochemical, hematological, coagulation, and inflammatory parameters between non-severe and severe cases is presented in Table 2. LDH, leucocyte, NLR, PTT, INR, fibrinogen, D-dimer, CRP, procalcitonin, and ferritin values were higher in the severe disease group. Patients with severe disease have shown significantly lower counts of lymphocytes and eosinophils.

**Figure 1:**
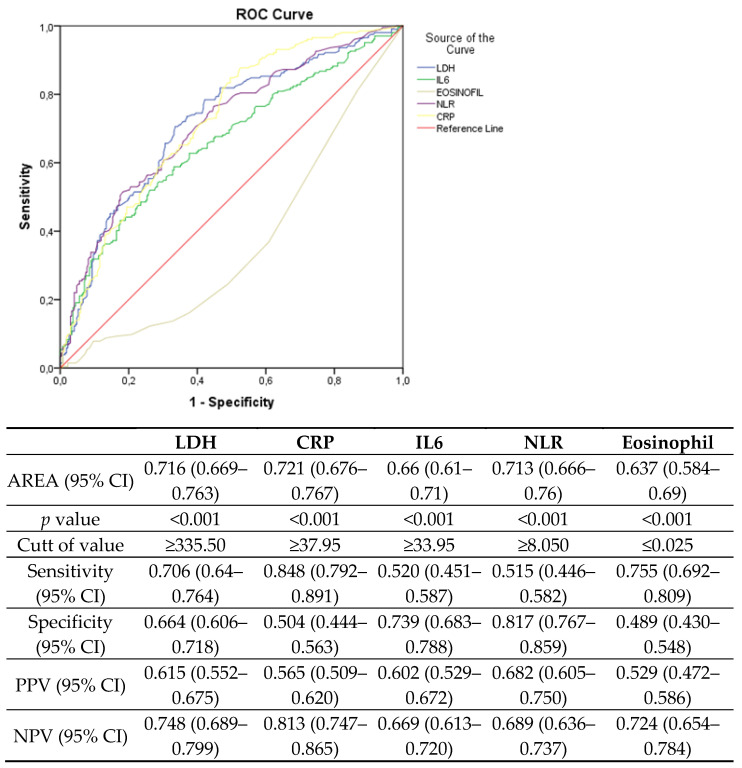
Receiving operator characteristic curve analysis of laboratory risk factors to predict severe disease in COVID-19 patients.

The inclusion of 27 variables in a multivariate logistic regression model resulted in 5 variables that were independently statistically significant predictors of severe illness, included in the new severity prediction score. These variables were CRP (OR, 2.681; 95% CI, 1.613–4.456; *p* < 0.001), eosinophil (OR, 1.959; 95% CI, 1.243–3.087; *p* = 0.004), NLR (OR, 2.785; 95% CI, 1.761–4.405; *p* < 0.001), IL-6 (OR, 2.008; 95% CI, 1.286–3.134; *p* = 0.002), and LDH (OR, 2.756; 95% CI, 1.778–4.724; *p* < 0.001) (Table 3). To facilitate clinical use and further assessment, a novel scoring model was established according to these predictive laboratory parameters, named CENIL (CRP, eosinophil, NLR, IL-6, LDH) scores from 0 to 5 points. Sensitivity, specificity, positive predictive, and negative predictive values were calculated for these significant predictive risk factors (Figure 1). For CRP, eosinophil, NLR, IL-6, and LDH, cut-off values were found as ≥37.95, ≤0.025, ≥8, ≥33.95, and ≥335.5, respectively.

**Figure 2:**
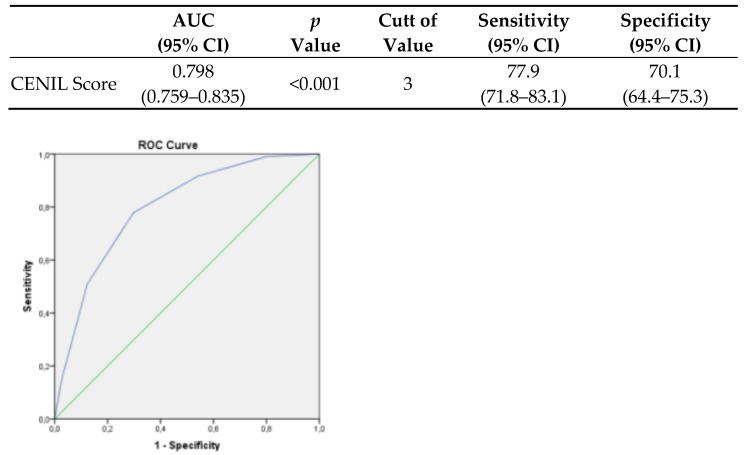
Receiver–operator characteristic curve of laboratory scoring system (CENIL SCORE) to predict severe disease in COVID-19 patients.

Receiver–operator curve (ROC) analysis was used to assess the performance of the CENIL scoring model, the area under the curve (AUC)(95% CI) was 0.798 (0.759–0.835) (Figure 2). When accepted cut-off values of 3 points, the sensitivity, specificity, positive and negative predictive value were 77.9% (71.8–83.1%), 70% (64.4–75.3%), 66.5% (60.3–72.2%), and 80.7% (75.1–85.2%), respectively (Table 4).

## 4. Discussion

The study found that a risk assessment score including laboratory parameters (CRP, eosinophil count, NLR, IL-6, and LDH-CENIL score) can help predict the likelihood of severe disease at diagnosis. A meta-analysis reviewed multiple studies and found that CRP, IL-6, NLR, and LDH levels consistently indicate severe COVID-19, supporting their use in clinical risk assessment models [15]. A study showed that eosinopenia is a significant predictor of COVID-19 severity, suggesting that low eosinophil levels upon admission are associated with worse outcomes in infected patients [16]. The multisystemic involvement in COVID-19 may include an overreactivated immune response that facilitates the severe disease [9]. Immune hyperactivation, known as a cytokine storm, leads to organ failure and has made these cytokines potential targets for COVID-19 treatment. Due to the urgent need, efforts have shifted toward repurposing existing drugs with known safety profiles to manage cytokine storms more quickly [17].

The SARS-CoV-2 has been shown in multiple tissues. The invasion of different tissues and organs may help explain the mechanism that causes the systemic effects of the virus. Therefore, laboratory biomarkers of organ damage can be used for the detection of patients at high risk of multiorgan involvement and severe disease [18]. Several biomarker scores are feasible in COVID-19. Some of these can predict disease prognosis and outcomes, while metabolomic and proteomic analysis parameters are still investigational concerns and difficult to apply in clinical practice [9]. We proposed a novel laboratory scoring system to aid in the prediction of disease severity for patients diagnosed with COVID-19. This score, which was based on biochemical, hematologic, inflammatory, and coagulation laboratory parameters, could be used and incorporated with clinical and radiological findings during the management of patients.

CRP is an acute phase reactant that was shown to be significantly elevated in the early stage of the disease, predicting severe COVID-19 [19]. The threshold cut-off value of CRP was found to be ≥40 mg/L as an early warning for close and cautious patient care by Stringer et al. They showed that elevated CRP value was indicative of severe disease and mortality [20]. The data presented here support these findings with a cut-off CRP value for severe disease of ≥37.95 mg/L. This value should be used to guide patients for clinical progressions in clinical practice.

IL-6 has strong proinflammatory effects and an important role in cytokine release syndrome. Elevated IL-6 levels have been observed in COVID-19 patients with pulmonary dysfunction, indicating cytokine-mediated pulmonary damage by SARS-CoV-2 viruses [21,22]. According to systematic reviews and meta-analyses, elevated IL-6 levels were found to be associated with adverse clinical outcomes and severity of COVID-19 [22,23]. We developed a new disease severity scoring system, including IL-6, which is not usually included in the scoring system.

Eosinopenia can be a predictor of disease severity among COVID-19 patients [24,25]. Cauchois et al. showed a significant association between severe eosinopenia and poor outcomes in hospitalized COVID-19 patients [26]. Eosinophil cell count was found to be lower in COVID-19 patients with cytokine storm [27]. Also, Denoël et al. demonstrated that eosinopenia was an independent risk factor of 30-day in-hospital mortality [28]. The current literature demonstrated that eosinophils could represent a possible biomarker for prognosis and prediction of disease severity in COVID-19 [24]. There were lots of scores based on biomarkers for COVID-19, but few of them included eosinopenia [28]. For example, Li et al. created a score combination of elevated CRP and eosinopenia to facilitate the triage of COVID-19 patients [29]. Eosinopenia was used as one of the components of the disease severity score in this study. This study can contribute to the literature on patient management in this manner.

NLR is considered a reliable and sensitive marker of immune activation and inflammation and interacts between innate and adaptive immunity [30]. The NLR value is a current prognostic biomarker in many diseases [31]. Also, this association was shown in COVID-19. Many studies found that a high NLR value was associated with a higher incidence of ARDS, higher rates of nonmechanical–mechanical ventilation, increased risk of severe disease, and one-month mortality in COVID-19 patients [32,33,34]. Smail et al. demonstrated that NLR was a predictor of oxygen saturation depression, a determining factor for disease severity [35]. Similarly, our data illustrated that NLR was an independent risk factor for severe COVID-19 disease.

LDH is an intracellular enzyme, and its concentration can be increased in severe infections by cytokine-mediated tissue damage [36]. Since LDH is present in lung tissue, severe COVID-19 patients can be expected to release higher amounts of LDH in the circulation. As a consequence of that, elevated LDH concentrations were found to be associated with a six-fold increase in developing severe disease in COVID-19 patients with pooled analysis [37]. Although most of the studies are retrospective and had high heterogenicity, He et al. showed that LDH concentrations were higher in severe COVID-19 in a systematic review and meta-analysis recently [38]. Similarly, increased LDH levels were statistically significantly higher in the severe group in this study.

Nowadays, risk prediction models and artificial neural networks are more common in medicine. Asteris et al. designed a risk prediction model for COVID-19 outcomes using artificial neural networks. This model included only five laboratory parameters: neutrophil-to-lymphocyte ratio, lactate dehydrogenase, fibrinogen, albumin, and D-dimer [39]. Also, our study includes only laboratory parameters (CRP, eosinophil, NLR, IL-6, LDH), which can be improved and applicable to clinical practice from this perspective.

This study presents potential limitations that should be acknowledged. First, the relatively small sample size may limit the robustness and generalizability of the developed risk score. Additionally, the study’s retrospective design might introduce biases and limit the temporal applicability of the findings, especially as the COVID-19 pandemic evolves with the emergence of new SARS-CoV-2 variants. For instance, research by Wang et al. indicated that inflammatory markers such as ferritin, LDH, and CRP were significantly lower in patients during the Omicron variant period, correlating with a milder disease course [40]. This variation suggests that our findings may not fully apply to patients infected with newer variants, and future studies should validate the score across different viral strains and cohorts. Moreover, while we included IL-6 in the scoring system due to its association with COVID-19 severity, this biomarker may not be universally accessible, as certain medical centers do not routinely measure it. Additionally, since our scoring system is based solely on laboratory parameters, it should ideally be used alongside clinical and radiological assessments to provide a comprehensive patient evaluation. Finally, to strengthen the broader implications of this study, future research should explore the score’s predictive value in diverse patient populations and settings, incorporating evolving clinical data. Expanding on these limitations, we encourage further validation studies to refine and adapt the risk-scoring system, enhancing its utility in dynamic healthcare contexts.

## 5. Conclusions

Early identification and targeted treatment of COVID-19 patients at risk of developing severe disease are essential. COVID-19 severity risk scoring systems have significant implications for clinical practice, aiding in the early identification of patients at risk for severe outcomes, which allows for prioritized treatment and resource allocation. For example, many of these models help clinicians predict patient outcomes such as ICU admission or mortality risk, thereby facilitating timely interventions (e.g., monitoring and ventilation). In this study, we examined the biochemical, hematological, coagulation, and inflammatory characteristics of COVID-19 patients to support personalized management. We developed a laboratory risk score, “CENIL”, based on these parameters, including CRP, eosinophil count, NLR, IL-6, and LDH. This scoring system integrates critical biomarkers, offering a reliable tool for predicting clinical outcomes and guiding therapeutic decisions.

## Figures and Tables

**Table 1:** Demographics and clinical characteristics of patients.

Characteristic	Non-Severe Group (*n* = 268)	Severe Group (*n* = 204)	Total Patients (*n* = 472)	*p*-Value
Age mean (±SD)	62.57 ± 15.92	66.25 ± 14.08	64 ± 3.1	0.009
Male *n* (%)	123 (45.9)	119 (58.3)	242 (51.3)	0.007
Comorbidity *n* (%)				
≥1 comorbidity	179 (66.8)	155 (76)	334 (70.7)	0.030
Hypertension	127 (47.4)	108 (52.9)	235 (49.8)	0.232
Diabetes mellitus	87 (32.5)	75 (36.8)	165 (35)	0.329
Coronary artery disease	49 (18.3)	51 (25)	100 (21.2)	0.077
COPD	24 (9)	41(20.1)	65 (13.8)	0.001
Hyperlipidemia	28 (10.4)	30 (14.7)	58 (12.3)	0.163
Chronic heart failure	15 (5.6)	16 (7.8)	31 (6.6)	0.329
Chronic kidney disease	18 (6.7)	12 (5.9)	30 (6.4)	0.713
Thyroid gland disease	14 (5.2)	12 (5.9)	26 (5.5)	0.756
Cerebrovascular disease	11 (4.1)	11 (5.4)	22 (4.7)	0.511
Abnormal Chest CT *n* (%)				
Unilateral involvement	45 (16.8)	11 (5.4)	56 (11.9)	˂0.001 ˂0.001
Bilateral involvement	178 (66.4)	191 (93.6)	369 (78.2)	
Corticosteroid *n* (%)	59 (22)	179 (87.7)	472 (58.9)	˂0.001
Oxygen support *n* (%)	65 (24.2)	204 (100)	348 (73.7)	˂0.001
ICU admission *n* (%)	9 (3.4)	79 (38.7)	98 (20.8)	˂0.001
Length of hospital stay median(min-max)	11 (1–46)	16 (2–57)	13 (1–57)	˂0.001
Mortality *n* (%)	0 (0)	26 (12.7)	26 (5.5)	˂0.001

COPD: chronic obstructive pulmonary disease; CT: computerized tomography; ICU: intensive care unit.

**Table 2:** Comparison of laboratory parameters between severe and non-severe COVID-19 patients.

	Non-Severe Patient (*n* = 268)	Severe Patient (*n* = 204)	Total Patient (*n* = 472)	*p*-Value
Biochemical parameters median (min–max)				
Glucose mg/dL	116 (66–421)	131 (63–441)	122 (63–441)	0.002
Creatinine mg/dL	0.84 (0.1–11)	0.93 (0.1–5)	0.9 (0.1–11)	0.015
Total protein g/L	63 (43–96)	61.5 (44–78)	63 (43–96)	<0.001
Albumin g/L	40 (19–50)	37 (25–46)	39 (19–50)	<0.001
CPK U/L	87.5 (11–6819)	127.5 (11–7600)	101.5 (11–7600)	<0.001
AST U/L	32 (4–257)	43.5 (6–412)	37 (4–412)	<0.001
ALT U/L	28 (3–285)	35 (7–383)	30 (3–383)	<0.001
Total bilirubin mg/dL	0.5 (0.1–5)	0.5 (0.1–3)	0.5 (0.1–5)	0.258
LDH U/L	283 (25–900)	388 (55–1180)	333 (25–1180)	<0.001
Troponin-I ng/L	5 (2–16,238)	10 (2–25,000)	7 (2–25,000)	<0.001
Hematologic biomarkers median (min–max)				
White blood cell × 10^9^/L	6 (1.35–20)	7 (2–21.5)	6.5 (1.35–21.5)	<0.001
Neutrophil count × 10^9^/L	4.2 (0.46–15.9)	5.8 (1.1–19.3)	4.8 (0.46–19.3)	<0.001
Lymphocyte count × 10^9^/L	0.97 (0.18–5)	0.69 (0.01–4)	0.89 (0.01–5)	<0.001
Eosinophil count × 10^9^/L	0.02 (0–0.49)	0.01 (0–0.53)	0.02 (0–0.53)	<0.001
Monocyte count × 10^9^/L	0.33 (0.03–1.6)	0.32 (0.02–1.5)	0.33 (0.02–1.6)	0.181
Hemoglobin g/dL	13.1 (7.8–17.2)	13.2 (7.8–17.5)	13.2 (7.8–17.5)	0.587
Platelet count × 10^9^/L	218 (77–589)	222 (29–720)	220 (29–720)	0.570
NLR	4.2 (0.7–36)	8.3 (1–84.3)	5.4 (0.7–84.3)	<0.001
Coagulation biomarkers median (min–max)				
aPTT s.	23.8 (17.6–55.8)	24.4 (16.7–55.9)	24 (16.7–55.9)	0.110
PTT s.	12.1 (9.9–39.2)	12.6 (9.6–39.1)	12.4 (9.6–39.2)	<0.001
INR	1.05 (0.3–3.5)	1.08 (0.8–3.5)	1.06 (0.3–3.5)	<0.001
Fibrinogen g/L	4.5 (0.8–10.1)	5.2 (1.5–10.1)	4.8 (0.8–10.1)	<0.001
D-dimer µg/ml	0.7 (0.1–35)	0.9 (0.2–35.2)	0.8 (0.1–35.2)	0.003
Inflammatory biomarkers median (min–max)				
CRP mg/L	37.7 (1–369)	99 (3–286)	60 (1–369)	<0.001
Procalcitonin µg/L	0.05 (0.01–11)	0.09 (0.02–9.7)	0.06 (0.01–11)	<0.001
Ferritin µg/L	233 (20–3413)	430 (26–5000)	308.5 (20–5000)	<0.001
IL-6 pg/mL	15.25 (1–350)	35.2 (2–2556)	19.9 (1–2556)	<0.001

CPK: creatinine phosphokinase; AST: aspartate transaminase; ALT: alanine transaminase; LDH: lactate dehydrogenase; NLR: neutrophil/lymphocyte ratio; aPTT: active partial thromboplastin time; PTT: prothrombin time; CRP: C-reactive peptide; IL-6: Interleukin-6.

**Table 3:** Multivariate logistic regression analysis of laboratory risk factors to predict severe disease in COVID-19 patients (*n* = 472).

	Univariate Analysis			Multivariate Analysis		
	OR	95% CI	*p*-Value	OR	95% CI	*p*-Value
CRP	5.665	3.608–8.893	<0.001	2.681	1.613–4.456	<0.001
Eosinophil	2.945	1.976–4.389	<0.001	1.959	1.243–3.087	0.004
NLR	4.740	3.134–7.171	<0.001	2.785	1.761–4.405	<0.001
IL-6	3.059	2.078–4.505	<0.001	2.008	1.286–3.134	0.002
LDH	4.747	3.202–7.031	<0.001	2.756	1.778–4.724	<0.001

**Table 4:** Laboratory scoring system (CENIL SCORE) to predict severe disease in COVID-19 patients.

CENIL Score	Sensitivity (95% CI)	Specificity (95% CI)	PPV (95% CI)	NPV (95% CI)
≥1	99 (96.5–99.7)	20.1 (15.8–25.4)	48.6 (43.8–53.4)	96.4 (87.9–99)
≥2	91.7 (87.1–94.7)	45.9 (0.40–51.9)	56.3 (50.9–61.6)	87.9 (81.4–92.3)
≥3	77.9 (71.8–83.1)	70.1 (64.4–75.3)	66.5 (60.3–72.2)	80.7 (75.1–85.2)
≥4	51 (44.2–57.8)	87.7 (83.2–91.1)	75.9 (68.1–82.3)	70.1 (65–74.8)
≥5	14.7 (10.5–20.2)	97.4 (94.7–98.7)	81.1 (65.8–90.5)	60 (55.3–64.5)

## Data Availability

All data needed to support the conclusions are present in the paper. Raw data are available from the corresponding author, E.M.S., upon reasonable request.

## References

[1] Sun D.-W., Zhang D., Tian R.-H., Li Y., Wang Y.-S., Cao J., Tang Y., Zhang N., Zan T., Gao L. (2020). The underlying changes and predicting role of peripheral blood inflammatory cells in severe COVID-19 patients: A sentinel?. Clin. Chim. Acta.

[2] Zhou F., Yu T., Du R., Fan G., Liu Y., Liu Z., Xiang J., Wang Y., Song B., Gu X. (2002). Clinical course and risk factors for mortality of adult inpatients with COVID-19 in Wuhan, China: A retrospective cohort study. Lancet.

[3] Cevik M., Bamford C.G.G., Ho A. (2020). COVID-19 pandemic—A focused review for clinicians. Clin. Microbiol. Infect..

[4] Ingraham N.E., Lotfi-Emran S., Thielen B.K., Techar K., Morris R.S., Holtan S.G., Dudley R.A., Tignanelli C.J. (2020). Immunomodulation in COVID-19. Lancet Respir. Med..

[5] Gao Y., Li T., Han M., Li X., Wu D., Xu Y., Zhu Y., Liu Y., Wang X., Wang L. (2020). Diagnostic utility of clinical laboratory data determinations for patients with the severe COVID-19. J. Med. Virol..

[6] Zhou R., Li F., Chen F., Liu H., Zheng J., Lei C., Wu X. (2020). Viral dynamics in asymptomatic patients with COVID-19. Int. J. Infect. Dis..

[7] Chen X., Zhao B., Qu Y., Chen Y., Xiong J., Feng Y., Men D., Huang Q., Liu Y., Yang B. (2020). Detectable serum severe acute respiratory syndrome coronavirus 2 viral load (RNAemia) is closely correlated with drastically elevated interleukin 6 level in critically ill patients with coronavirus disease 2019. Clin. Infect. Dis..

[8] Wang L. (2020). C-reactive protein levels in the early stage of COVID-19. Med. Mal. Infect..

[9] Statsenko Y., Al Zahmi F., Habuza T., Gorkom K.N.-V., Zaki N. (2021). Prediction of COVID-19 severity using laboratory findings on admission: Informative values, thresholds, ML model performance. BMJ Open.

[10] World Health Organization (2020). Clinical Management of Severe Acute Respiratory Infection (SARI) When COVID-19 Disease Is Suspected: Interim Guidance, 13 March 2020. World Health Organization..

[11] Battaglini D., Lopes-Pacheco M., Castro-Faria-Neto H.C., Pelosi P., Rocco P.R.M. (2022). Laboratory biomarkers for diagnosis and prognosis in COVID-19. Front. Immunol..

[12] Birlutiu V., Neamtu B., Birlutiu R.-M. (2024). Identification of Factors Associated with Mortality in the Elderly Population with SARS-CoV-2 Infection: Results from a Longitudinal Observational Study from Romania. Pharmaceuticals.

[13] Skakun O., Vandzhura Y., Vandzhura I., Symchych K., Symchych A. (2024). Biomarkers for unfavourable outcomes prediction in COVID-19 patients: A narrative review. J. Emerg. Crit. Care Med..

[14] Salton F., Confalonieri P., Campisciano G., Cifaldi R., Rizzardi C., Generali D., Pozzan R., Tavano S., Bozzi C., Lapadula G. (2022). Cytokine Profiles as Potential Prognostic and Therapeutic Markers in SARS-CoV-2-Induced ARDS. J. Clin. Med..

[15] Henry B.M., de Oliveira M.H.S., Benoit S., Plebani M., Lippi G. (2020). Hematologic, biochemical and immune biomarker abnormalities associated with severe illness and mortality in coronavirus disease 2019 (COVID-19): A meta-analysis. Clin. Chem. Lab. Med..

[16] Lippi G., Henry B.M. (2020). Eosinophil count in severe coronavirus disease 2019. Qjm: Int. J. Med..

[17] Agresti N., Lalezari J.P., Amodeo P.P., Mody K., Mosher S.F., Seethamraju H., Kelly S.A., Pourhassan N.Z., Sudduth C.D., Bovinet C. (2021). Disruption of CCR5 signaling to treat COVID-19-associated cytokine storm: Case series of four critically ill patients treated with leronlimab. J. Transl. Autoimmun..

[18] Robba C., Battaglini D., Pelosi P., Rocco P.R.M. (2020). Multiple organ dysfunction in SARS-CoV-2: MODS-CoV-2. Expert Rev. Respir. Med..

[19] Tan C., Huang Y., Shi F., Tan K., Ma Q., Chen Y., Jiang X., Li X. (2020). C-reactive protein correlates with computed tomographic findings and predicts severe COVID-19 early. J. Med. Virol..

[20] Stringer D., Braude P., Myint P.K., Evans L., Collins J.T., Verduri A., Quinn T.J., Vilches-Moraga A., Stechman M.J., Pearce L. (2021). The role of C-reactive protein as a prognostic marker in COVID-19. Int. J. Epidemiol..

[21] Jones S.A., Jenkins B.J. (2018). Recent insights into targeting the IL-6 cytokine family in inflammatory diseases and cancer. Nat. Rev. Immunol..

[22] Zhu J., Pang J., Ji P., Zhong Z., Li H., Li B., Zhang J. (2021). Elevated interleukin-6 is associated with severity of COVID-19: A meta-analysis. J. Med Virol..

[23] Zhu J., Pang J., Ji P., Zhong Z., Li H., Li B., Zhang J. (2020). Interleukin-6 in COVID-19: A systematic review and meta-analysis. J. Med. Virol..

[24] Macchia I., La Sorsa V., Urbani F., Moretti S., Antonucci C., Afferni C., Schiavoni G. (2023). Eosinophils as potential biomarkers in respiratory viral infections. Front. Immunol..

[25] Chen D., Zhang S., Feng Y., Wu W., Chang C., Chen S., Zhen G., Yi L. (2021). Decreased eosinophil counts and elevated lactate dehydrogenase predict severe COVID-19 in patients with underlying chronic airway diseases. Postgrad. Med. J..

[26] Cauchois R., Pietri L., Dalmas J.-B., Koubi M., Capron T., Cassir N., Potere N., Polidoro I., Jean R., Jarrot P.-A. (2022). Eosinopenia as Predictor of Poor Outcome in Hospitalized COVID-19 Adult Patients from Waves 1 and 2 of 2020 Pandemic. Microorganisms.

[27] Koc I., Ozmen S.U. (2022). Eosinophil levels, neutrophil-lymphocyte ratio, and platelet-lymphocyte ratio in the cytokine storm period of patients with COVID-19. Int. J. Clin. Pract..

[28] Denoël P., Brousmiche K., Castanares-Zapatero D., Manara A., Yombi J.C. (2023). Role of Eosinopenia as a Prognostic Factor in COVID-19 Patients from Emergency Department During the Second Wave. SN Compr. Clin. Med..

[29] Li Q., Ding X., Xia G., Chen H.-G., Chen F., Geng Z., Xu L., Lei S., Pan A., Wang L. (2020). Eosinopenia and elevated C-reactive protein facilitate triage of COVID-19 patients in fever clinic: A retrospective case-control study. EClinicalMedicine.

[30] Zahorec R. (2021). Neutrophil-to-lymphocyte ratio, past, present and future perspectives. Bratisl. Med. J..

[31] Keykavousi K., Nourbakhsh F., Abdollahpour N., Fazeli F., Sedaghat A., Soheili V., Sahebkar A. (2022). A Review of Routine Laboratory Biomarkers for the Detection of Severe COVID-19 Disease. Int. J. Anal. Chem..

[32] Ma A., Cheng J., Yang J., Dong M., Liao X., Kang Y. (2020). Neutrophil-to-lymphocyte ratio as a predictive biomarker for moderate-severe ARDS in severe COVID-19 patients. Crit. Care.

[33] Moradi E.V., Teimouri A., Rezaee R., Morovatdar N., Foroughian M., Layegh P., Kakhki B.R., Koupaei S.R.A., Ghorani V. (2020). Increased age, neutrophil-to-lymphocyte ratio (NLR) and white blood cells count are associated with higher COVID-19 mortality. Am. J. Emerg. Med..

[34] Waris A., Din M., Khalid A., Lail R.A., Shaheen A., Khan N., Nawaz M., Baset A., Ahmad I., Ali M. (2021). Evaluation of hematological parameters as an indicator of disease severity in COVID-19 patients: Pakistan’s experience. J. Clin. Lab. Anal..

[35] Smail S.W., Babaei E., Amin K. (2023). Hematological, Inflammatory, Coagulation, and Oxidative/Antioxidant Biomarkers as Predictors for Severity and Mortality in COVID-19: A Prospective Cohort-Study. Int. J. Gen. Med..

[36] Martinez-Outschoorn U.E., Prisco M., Ertel A., Tsirigos A., Lin Z., Pavlides S., Wang C., Flomenberg N., Knudsen E.S., Howell A. (2011). Ketones and lactate increase cancer cell “stemness,” driving recurrence, metastasis and poor clinical outcome in breast cancer. Cell Cycle.

[37] Henry B.M., Aggarwal G., Wong J., Benoit S., Vikse J., Plebani M., Lippi G. (2020). Lactate dehydrogenase levels predict coronavirus disease 2019 (COVID-19) severity and mortality: A pooled analysis. Am. J. Emerg. Med..

[38] He Z., Yan R., Liu J., Dai H., Zhu Y., Zhang F., Zhang L., Yan S. (2023). Lactate dehydrogenase and aspartate aminotransferase levels associated with the severity of COVID-19: A systematic review and meta-analysis. Exp. Ther. Med..

[39] Asteris P.G., Kokoris S., Gavriilaki E., Tsoukalas M.Z., Houpas P., Paneta M., Koutzas A., Argyropoulos T., Alkayem N.F., Armaghani D.J. (2023). Early prediction of COVID-19 outcome using artificial intelligence techniques and only five laboratory indices. Clin. Immunol..

[40] Wang J., Choy K.W., Lim H.Y., Ho P. (2022). Laboratory markers of severity across three COVID-19 outbreaks in Australia: Has Omicron and vaccinations changed disease presentation?. Intern. Emerg. Med..

